# USING STORYTELLING TO ASSESS NURSE’S KNOWLEDGE IN CARING FOR OLDER ADULTS

**DOI:** 10.1093/geroni/igac059.3131

**Published:** 2022-12-20

**Authors:** Michele Murphy-Boucher, Diana Lynn Woods

**Affiliations:** Providence Holy Cross Medical Center/Azusa Pacfic University, Mission Hills, California, United States; Azusa Pacific University, Azusa, California, United States

## Abstract

Continuing education, knowledge acquisition, and competence is required of all professional Registered Nurses. Remaining current in care delivery trends based on evidence practice is the responsibility of nurses supported by professional development specialists and educators. This project was conducted as a quality improvement project, using the evidence-based teaching modality of storytelling to educate practitioners in caring for older adults. This project addresses the question: For leaders of professional development and education focused on acute care and ambulatory practice, who have not had specialty geriatric training, how does using case-based/story-telling education for the care of patients 65 years and older affect confidence levels of the 4 M’s (mentation, mobility, what matters and medication) of elder care? A convenience sample of 12 Directors of Professional Development participated, and a validated comparison of responses to the Gerontological Nursing Competence Questionnaire (GNCQ) and the Facts on Aging (2015 version) (FAQ) were used for collecting data of pre intervention and post intervention. An eight-minute video was the educational intervention. GNCQ responses for confidence in knowledge and confidence in teaching were statistically significant. Although an increase in the mean score for interest in additional training, it was not statistically significant. The FAQ showed no statistical significance for the pre and post results. Findings indicate that storytelling is a viable teaching modality for increasing nurse’s knowledge of caring for older adults based on the concepts of the 4 Ms of age-friendly care. Keywords: storytelling, case studies, nurse education, professional development, narratives, and adult learning.

